# Supplementary figures and images for: Systematic analysis of cilia characteristics and Hedgehog signaling in five immortal cell lines
Source: PLoS One. 2022 Dec 29;17(12):e0266433. doi: 10.1371/journal.pone.0266433 (PMC9799305; doi:10.1371/journal.pone.0266433)

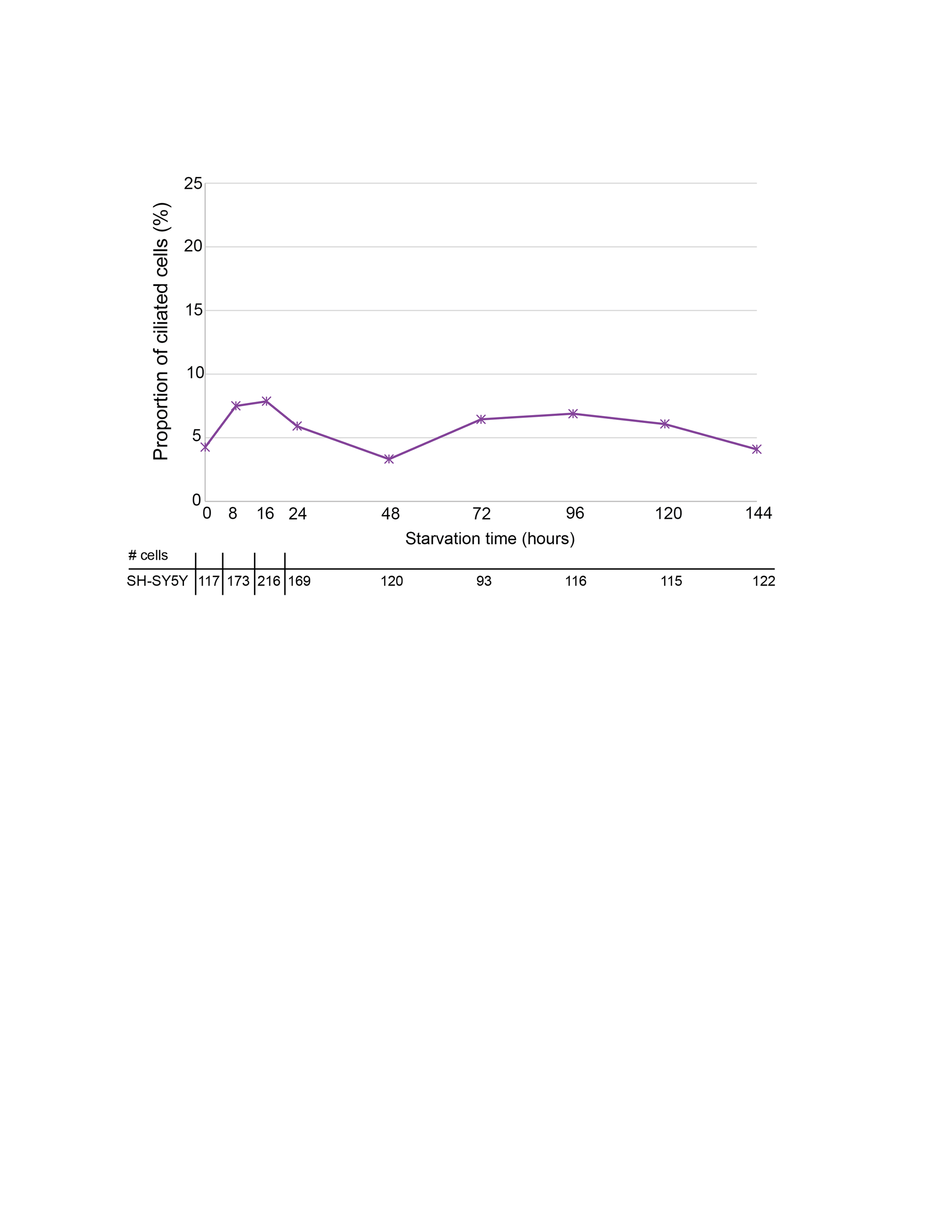

Supplement: S1 Fig — Proportion of ciliated cells for SH-SY5Y cells from 0–144 hours. One coverslip was imaged for each timepoint, with number of cells counted at each time point listed below each column in the graph. (TIF) [file pone.0266433.s001.tif]

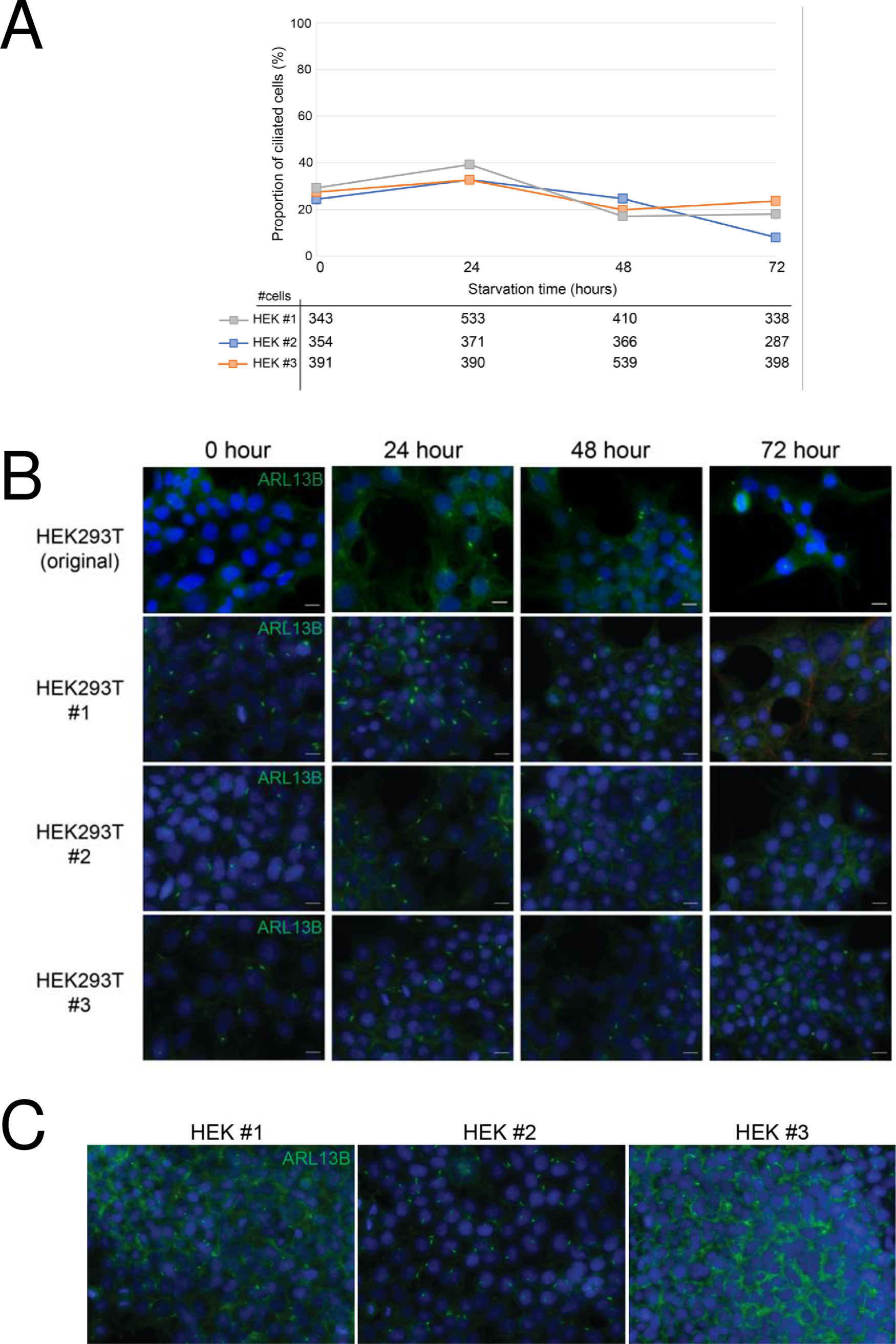

Supplement: S2 Fig — A) Proportion of ciliated HEK293T cells at 0, 24, 48, and 72 hours post serum starvation from additional thaws. B) Representative images of monolayer areas of HEK293T cell growth from Fig 1, Batch 3, which had the highest ciliation rates (top row) and repeat experiments with additional thaws of HEK293T cells. Cilia were stained with anti-ARL13B antibody. C) Representative images displaying ciliation in areas with multi-layer growth from additional thaws of HEK293T cells. Cilia were stained with anti-ARL13B antibody. Scale bars are 10μm. (TIF) [file pone.0266433.s002.tif]

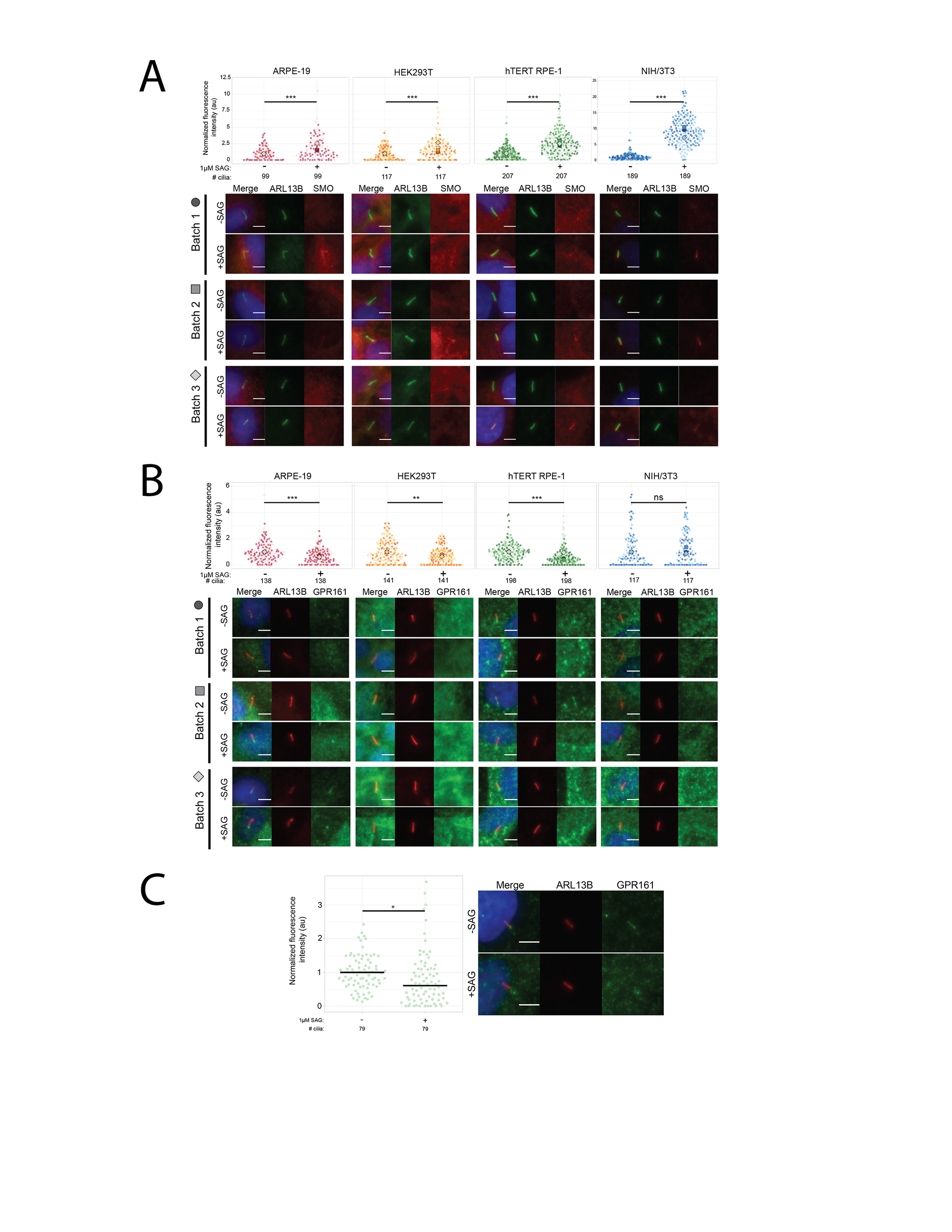

Supplement: S3 Fig — Representative images from each batch showing variation in A) SMO and B) GPR161 localization. C) Normalized fluorescence intensity (arbitrary units, au) of GPR161 with and without stimulation in an additional passage of hTERT RPE-1 cells. Black bars = median normalized fluorescence intensity. Small symbol = normalized fluorescence intensity of one cilium. Numbers of cilia measured are listed below each column in the graph. Representative cilia are shown next to the graphs for unstimulated (-SAG) and stimulated (+SAG) cells. Scale bars are 5 μm. T-test of equal variances was performed. p-values: * 0.01 to 0.05, ** 0.001 to 0.01, and *** <0.001. (TIF) [file pone.0266433.s003.tif]

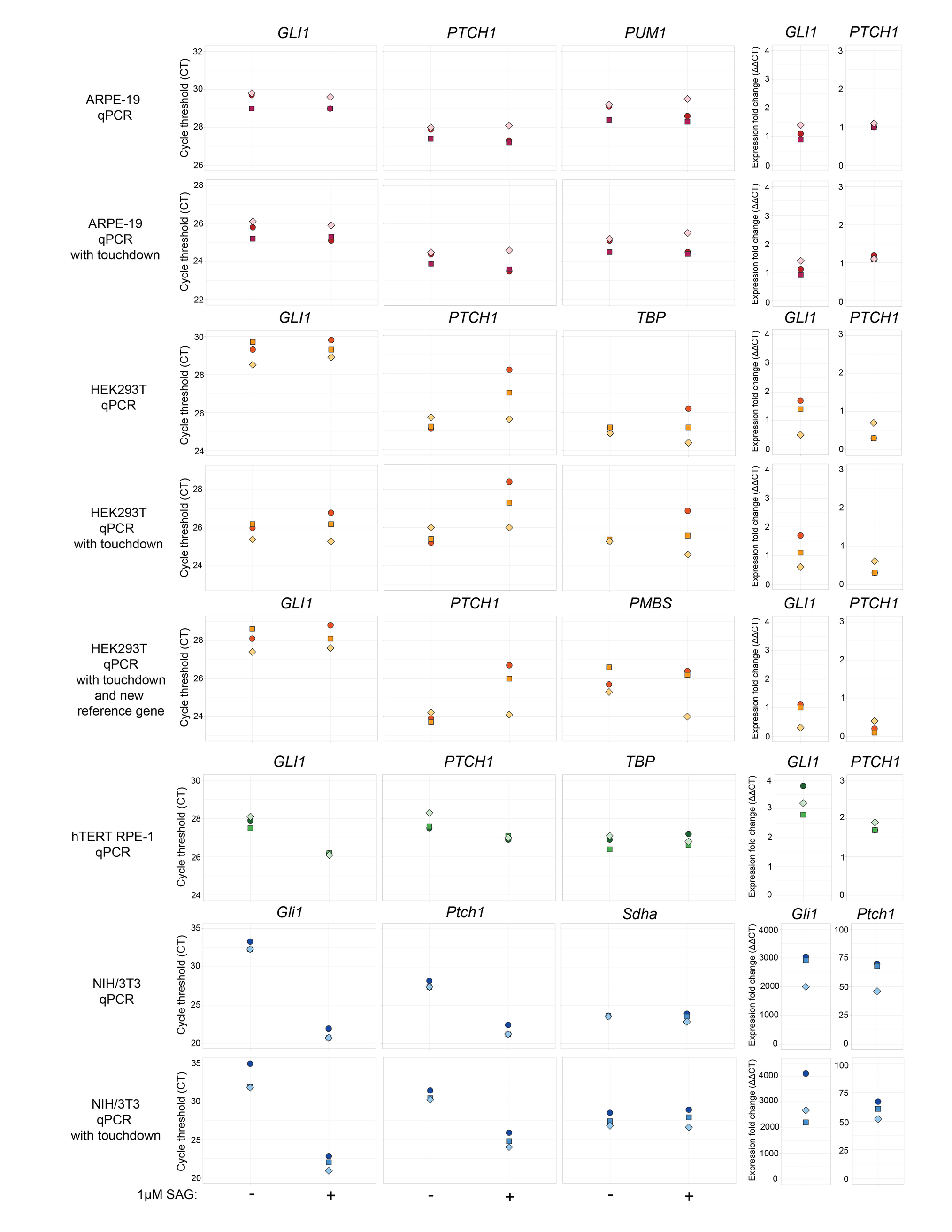

Supplement: S4 Fig — GLI1, PTCH1, and reference gene CT (cycle threshold) for ARPE-19, HEK293T, hTERT RPE-1, and NIH/3T3 cell lines with and without stimulation. For cell lines with high CT values (>29), we used a touchdown qPCR method. For each condition (with or without stimulation), we collected mRNA from three batches for each cell line. Normalized GLI1 and PTCH1 expression for each qPCR condition is on the far right. Each symbol in the left panels represents the average CT from three technical replicates for each batch. Each symbol in the right panels represents the expression fold change for each batch. (TIF) [file pone.0266433.s004.tif]
